# Gelation as a Dynamical Instability of the Smoluchowski Flow

**DOI:** 10.1021/acs.jpcb.6c01583

**Published:** 2026-04-14

**Authors:** Manuel Dedola, Ludovico Cademartiri

**Affiliations:** Department of Chemistry, Life Sciences and Environmental Sustainability, 9370 University of Parma, Parco Area delle Scienze 17 a, Parma 43124, Italy

## Abstract

Gelation is commonly interpreted as a finite-time singularity in the Smoluchowski coagulation equation marked by mass loss or moment divergence. We instead characterize gelation as a loss of dynamical stability of the Smoluchowski flow, quantified through the time-dependent spectrum of the Jacobian along the evolving aggregation dynamics. Studying homogeneous kernels *K*(*i,j*) = (*ij*)^α^ together with the classical Smoluchowski, we show that gelation is consistently preceded by the appearance of positive real eigenvalues, indicating a loss of local dynamical stability. While nongelling kernels exhibit only transient finite-size effects, gelling kernels display persistent spectral destabilization associated with macroscopic gel formation. These results place gelation within a unified dynamical framework of aggregation-driven phase transitions by identifying a spectral signature that links the kinetic runaway of gelation to the metastability of nucleation in Becker–Döring kinetics.

## Graphical abstract

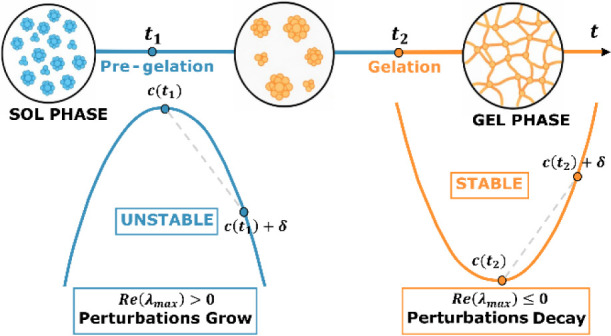

## Introduction

The Smoluchowski coagulation equation is a cornerstone model for irreversible aggregation processes, with applications ranging from colloidal aggregation and polymerization to cloud formation and network growth.^1,2^ It describes the time evolution of the cluster size distribution through binary coagulation events governed by an interaction kernel *K*(*i,j*) that encodes the rate at which clusters of sizes *i* and *j* merge.

One of the most striking phenomena predicted by Smoluchowski’s coagulation equations is *kinetic* gelation, namely the formation of a macroscopic cluster that absorbs a finite fraction of the total mass in finite time. We emphasize that this kinetic runaway growth—distinct from density-driven jamming or percolation^3^—is an intrinsic property of the aggregation kernel in the dilute limit, rather than a structural arrest triggered primarily by crossing a critical volume fraction like gelation in attractive colloidal suspensions or dense granular media.^4^ Furthermore, in physical colloidal systems, the structure and onset of gelation are heavily influenced by the interparticle attraction energy. As demonstrated in studies linking master kinetic equations to colloidal rheology, the attraction strength governs bond lifetimes and interaction-driven assembly pathways.^5^ The standard Smoluchowski framework investigated here corresponds to the limit of strong attraction (irreversible aggregation with infinite bond lifetimes).

Since Smoluchowski’s original work,^1^ gelation has traditionally been characterized through moment-based criteria, such as the divergence of the second and higher-order moments—representing the rapid increase in mean cluster size and polydispersity—or the loss of mass conservation (i.e., the formation of a separate gel phase) beyond a critical time *t*_*g*_.^3,7–10^ For instance, in the case of homogeneous kernels of the form *K*(*i,j*) = (*ij*)^α^, classical theory predicts gelation for $\alpha >\frac{1}{2}$ , while subcritical kernels remain mass conserving for all times.^3,11,12^

Modern interpretations increasingly describe gelation as a nonequilibrium continuous (second-order) phase transition governed by a percolation process. Within this framework, gelation corresponds to a transition where cluster size and correlation length exhibit critical scaling, with power-law divergences characterized by critical exponents (e.g., ∼1.6 and ∼0.8, respectively, for 3D percolation). Furthermore, when detailed balance is broken—meaning particle attachment and detachment rates are asymmetric—the cluster mass distribution follows universal power laws, scaling as *k* ^–3/2^ in the sol phase and *k* ^–5/2^ in the gel phase.^13,14^

In this work, we adopt a complementary perspective by focusing on the local dynamical stability of the aggregation process. While the critical-phenomena perspective successfully captures the universal, asymptotic scaling behavior associated with gelation, it primarily provides a statistical description of the transition. In contrast, our approach interprets gelation as the onset of a dynamical instability detected through the Jacobian spectrum of the Smoluchowski flow. The emergence of eigenvalues with positive real parts signals a loss of local stability, providing a time-resolved criterion that links macroscopic arrest to the underlying dynamics of the aggregation flow.

Despite this well-established theoretical picture, the dynamical nature of gelation remains conceptually subtle. The description of gelation as divergent moments or the introduction of a separate gel phase,^3^ while mathematically precise, provides limited insight into the *collective mechanism* by which it occurs. Moreover, numerical studies have long reported finite-size effects, transient mass loss, and apparent gelation-like behavior even for kernels that are theoretically nongelling.^15^

This raises a fundamental question: *Is gelation merely a singularity of moments, or does it also correspond to a genuine dynamical instability?*

In this work, we address this question from a dynamical systems perspective. Rather than focusing on global observables such as mass or moments (which diagnose gelation “after the fact”), we analyze the *local time-resolved stability* of the full Smoluchowski coagulation equations, viewed as an infinite-dimensional nonlinear ODE system.

Physically, the time evolution of the cluster size distribution in composition space is the flow of the Smoluchowski coagulation equations. A dynamical instability of this flow indicates a regime in which there exist infinitesimal perturbations to the population—whether from thermal fluctuations, discreteness, or finite-size effects—that are systematically amplified rather than damped, leading to runaway growth and macroscopic structure formation. In this sense, gelation could be then interpreted as the point at which the mean-field aggregation dynamics ceases to be locally self-stabilizing.

The Jacobian is the most convenient way to quantify the local response and stability of the Smoluchowski flow to perturbations as its eigenvalues are the growth rates of the fluctuations. Although the Smoluchowski coagulation equations admit no nontrivial steady states prior to gelation, their time-dependent flow is well-defined, and the instantaneous Jacobian spectrum provides time-resolved information on the amplification or decay of perturbations. The emergence of eigenvalues with positive real parts signals a loss of local stability, independently of moment divergence or mass conservation.

We apply this framework to homogeneous multiplicative kernels in both subcritical and supercritical regimes, to the classical Smoluchowski kernel, and to a nongelling additive kernel used as a control case. Our results show that gelation is consistently *preceded* by a persistent spectral destabilization of the Jacobian, while nongelling kernels exhibit at most transient, finite-size instabilities that vanish in the large-cutoff limit.

These findings provide strong evidence that gelation should be interpreted not only as a singularity of moments, but as the manifestation of a *dynamical instability of the aggregation flow itself*. By reframing gelation in terms of stability theory, this work establishes a direct conceptual link between classical coagulation theory and modern dynamical systems analysis, and introduces a robust diagnostic tool for identifying gelation beyond traditional moment-based criteria. Interestingly, a closely related spectral criterion is known to govern the onset of nucleation in the reversible Becker–Döring equations, hinting at a deep dynamical commonality between aggregation-driven phase transitions.

### Model and Stability Framework

We consider the discrete Smoluchowski coagulation equation for the cluster size distribution *c_P_*:^16^

$${ċ}_{p}=\frac{1}{2}\underset{i+j=p}{\sum }K_{ij}c_{i}c_{j}-c_{p}\overset{\infty }{\underset{j=1}{\sum }}K_{pj}c_{j}$$ 1

Where *c_P_* (*t*) denotes the concentration of clusters of size *p* at time *t*, and *K_ij_* is the coagulation kernel. Throughout this work, concentrations are measured in units of number density, and time is rescaled so that the overall collision rate is unity.

### Kernels Considered

We use two representative classes of coagulation kernels to test the robustness of the spectral stability analysis.

#### Multiplicative Homogeneous Kernels

In order to test the consistency of our stability analysis with classical theory we employ this class of kernels expressed as

2 $$K_{ij}={\left(ij\right)}^{\alpha }$$

This well-known class exhibits a sharp transition in the kinetic behavior: classical theory predicts gelation (i.e., mass loss in finite time) for $\alpha >\frac{1}{2}$ , while mass is conserved for $\alpha \leq \frac{1}{2}$ .^4,7^ This allows us to sweep across the critical threshold and monitor the Jacobian spectrum.

#### Smoluchowski Diffusion-Limited Kernel

As a physically motivated nongelling control, we consider the classical kernel for Brownian coagulation of fractal aggregates in the continuum regime:^17^

3 $$K\left(i,j\right)=\frac{1}{4}\left(i^{1/D_{f}}+j^{1/D_{f}}\right)\left(i^{-\frac{1}{D_{f}}}+j^{-\frac{1}{D_{f}}}\right)$$

where *D_f_* represents the fractal dimension of the aggregates. In our simulations, we fix *D_f_* = 1.8, a typical value for diffusion-limited cluster aggregation (DLCA).^18^ The Smoluchowski kernel is homogeneous (*K*(Λ*i*,Λ*j*) = Λ*^ω^K*(*i,j*)) of degree ω = 0. According to the rigorous classification by van Dongen and Ernst,^3,11^ homogeneous kernels with ω ≤ 1 obey mass conservation for all times and do not undergo kinetic gelation.

### Finite-Dimensional Truncation

To make the problem numerically tractable, we introduce a cutoff *p* and restrict cluster sizes to 1 ≤ *i* ≤ *p*. This yields a finite-dimensional nonlinear ODE system,

4 $$ċ=F\left(t\right);\;c\left(t\right)\in R^{p}$$

which converges to the infinite-dimensional dynamics as *p* → ∞ prior to gelation. All numerical results are obtained by systematically increasing *p* to assess finite-size effects.

### Linearization along the Trajectory

Unlike classical stability analyses based on fixed points, the Smoluchowski coagulation equations admit no nontrivial steady states in the pregelation regime. We therefore adopt a *time-dependent linearization* approach. Given a solution ***c*** (***t***) of eq 1 we define the Jacobian matrix:^19,20^

$$J\left(t\right)={\frac{\partial F}{\partial c}]}_{c\left(t\right)}$$ 5

which governs the evolution of infinitesimal perturbations *δ**c*** (***t***) via:

6 $$\frac{d}{dt}\delta c=J\left(t\right)\delta c$$

At each time ***t***, the spectrum of ***J*** (***t***) provides instantaneous information on the local stability of the flow. While the Jacobian of the aggregation is non–normal—meaning perturbation could theoretically grow for a short time even if the system is stable—the persistent increase and appearance of positive eigenvalues provide a clear signal that the system has become fundamentally unstable.

### Explicit Jacobian Form

The linearization of the discrete Smoluchowski coagulation equation yields the exact analytical form of the Jacobian operator elements *J_ij_*:

7 $$J_{ij}\left(t\right)=\frac{\partial {ċ}_{i}}{\partial c_{j}}=K_{j,i-j}c_{i-j}\left(t\right)I_{j<i}-K_{ij}c_{i}\left(t\right)-\delta_{ij}\overset{p}{\underset{k=1}{\sum }}K_{ik}c_{k}\left(t\right)$$

where *δ_ij_* is the Kronecker delta and $I_{j<i}$ is the indicator function, which equals 1 if the condition is met and 0 otherwise. We emphasize that the Jacobian spectrum is evaluated along the trajectory ***c*** (*t*) and therefore does not define asymptotic stability in the strict dynamical systems sense. However, the persistence of positive real eigenvalues over extended time intervals provides a robust indicator of a genuine dynamical destabilization of the flow.

### Spectral Indicator of Gelation

Unlike classical stability analyses that rely on stationary fixed points, the Smoluchowski coagulation equation defines a time-dependent, nonautonomous flow that admits no nontrivial steady states prior to gelation. Because the system is constantly evolving, classical asymptotic Lyapunov exponents are not defined. This distinct feature requires us to shift from a fixed-point stability approach to a trajectory-based one.

To understand this intuitively, one can imagine the concentration space as a multidimensional landscape shaped by the interaction rates. The aggregation dynamics describe a trajectory—the specific path the system takes across this landscape over time. Because we cannot evaluate stability at a resting point, we must evaluate the instantaneous linear behavior of the system at our current location along the trajectory.

We therefore adopt a local (finite time) Lyapunov framework, in which stability is quantified through the instantaneous Jacobian spectrum evaluated along the evolving trajectory. The Jacobian matrix, J­(t), acts as a local topographical map: its eigenvalues determine whether the system is locally self-stabilizing or prone to runaway growth. In particular, the maximum real part of the Jacobian eigenvalues:

8 $$\lambda_{max}\left(t\right)=\max\left\{Re\left(\lambda \right):\lambda \in \sigma \left(J\left(t\right)\right)\right\}$$

represents a local Lyapunov growth rate, characterizing the instantaneous amplification or decay of infinitesimal perturbations. In chemical terms, if Re­(λ_max_) is negative, perturbations decay, and the trajectory is locally stable. If positive real eigenvalues appear, it indicates an exponential growth of fluctuations (instability) along that specific direction in the concentration space.

In this framework, a persistent positive λmax­(*t*) provides a robust diagnostic for genuine gelation, signaling a sustained dynamical destabilization of the flow that is independent of mass conservation or moment divergence. This criterion successfully distinguishes physical instabilities from transient finite-size artifacts, which vanish as the system cutoff increases. Our results show that gelation is reframed as an intrinsic dynamical instability of the aggregation flow itself (Figure 1).

**Figure 1:**
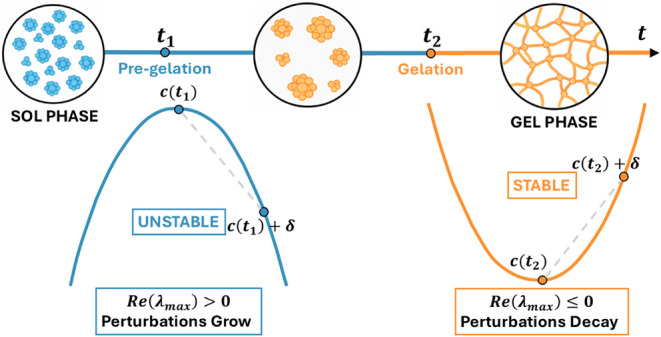
Structural and dynamical evolution during gelation. The transition from the Sol phase (dispersed clusters) to the Gel phase (macroscopic network) is accompanied by a shift in stability. The system evolves from a linearly unstable state (Re­(λ_max_)) at *t*_1_ to a stable configuration at *t*_2_.

## Computational Design

The numerical framework was developed to solve the truncated Smoluchowski system and track its spectral evolution in real time.

### Numerical Integration and Truncation

We implement the discrete Smoluchowski coagulation equation by introducing a finite-size cutoff *p*, restricting cluster sizes to 1 ≤ *i* ≤ *p*. The resulting system of *p* nonlinear ODEs is integrated using the *MATLAB* ODE solver (*ode15s*).

The system is initialized as a monodisperse suspension, *c*_1_(0) = 1, with concentrations normalized by the initial total number density, *c_n_* = *N_n_*/*N*_0_. The dynamics are integrated in rescaled, dimensionless time τ. At each integration step, the coagulation rates *K_ij_* are precomputed as a *p* × *p* matrix to optimize the evaluation of the gain and loss terms.

### Comparative Scaling and Convergence

To differentiate between physical gelation and numerical artifacts, the simulation protocol includes:

### Kernel Comparison

Systematic testing of gelling multiplicative kernels (α > 1/2) against nongelling additive and diffusion-limited controls.

### Cutoff Scaling

To distinguish genuine dynamical features from finite-size artifacts, we performed a scaling analysis by varying the system size cutoff *p* (e.g., from 500 to 2000). This ensures that the observed spectral destabilization is a persistent feature of the infinite-dimensional flow rather than a transient effect of the truncation. Based on the convergence analysis (see Supporting Information, Figure S1), we set *p* = 1000 for the main results, as this value was found sufficient to capture the asymptotic behavior of the sol–gel transition while maintaining computational efficiency.

### Spectra Implementation

Unlike traditional postprocessing, our algorithm computes the Jacobian matrix ***J*** (*t*) at each discrete time step *t_n_* along the trajectory. The matrix ***J*** is constructed by explicitly evaluating the partial derivatives $\frac{\partial {ċ}_{i}}{\partial c_{j}}$ for the current concentration vector ***c*** (***t***).

We perform a full eigen decomposition to extract the spectrum σ­(***J*** (***t***)). This allows for the simultaneous tracking of the dominant growth rate Re­(λ_max_) and the overall distribution of eigenvalues in the complex plane.

### Observables and Statistical Indicators

To complement the spectral analysis, the framework tracks the evolution of global observables that characterize the aggregation state and the distribution’s complexity:

Moments Analysis: We compute the first moment $\left(M_{1}=\overset{p}{\underset{i=1}{\sum }}ic_{i}\right)$ to monitor total mass conservation and identify the critical time *t_g_* when mass loss occurs in supercritical regimes. Simultaneously, the second moment $\left(M_{2}=\overset{p}{\underset{i=1}{\sum }}i^{2}c_{i}\right)$ is tracked as the standard indicator of polydispersity, signaling the traditional gelation singularity through its divergence.
Shannon Entropy (H): To quantify the informational evolution of the cluster size distribution, we calculate the instantaneous Shannon entropy:
$$H\left(t\right)=-\overset{p}{\underset{i=1}{\sum }}P_{i}\ln P_{i}$$

where $P_{i}=c_{i}/\sum c_{i}$ represents the normalized probability of finding a cluster of size *i*. This metric provides a measure of the “disorder” of the distribution as it shifts from a monodisperse monomer state to a polydisperse sol and, eventually, to the gel phase.

## Results and Discussion

We first examine the dynamical stability of the aggregation process by comparing two representative multiplicative kernels, *K*(*ij*) = (*ij*)^α^, in the subcritical (α = 0.2) and supercritical (α = 0.8) regimes.

In the nongelling regime (α = 0.2), the system remains in the sol phase indefinitely. As shown in Figure 2 (panels corresponding to α= 0.2), the first moment *M*_1_ is strictly conserved, and the second moment *M*_2_ grows monotonically without divergence. The maximum real eigenvalue of the Jacobian remains vanishingly small (Re­(λ_max_)) ≈ 0) throughout the evolution. This behavior reflects the neutral stability inherent to the Smoluchowski flow under mass-conserving kernels and indicates that perturbations introduced into the sol phase decay or propagate without amplification.

**Figure 2:**
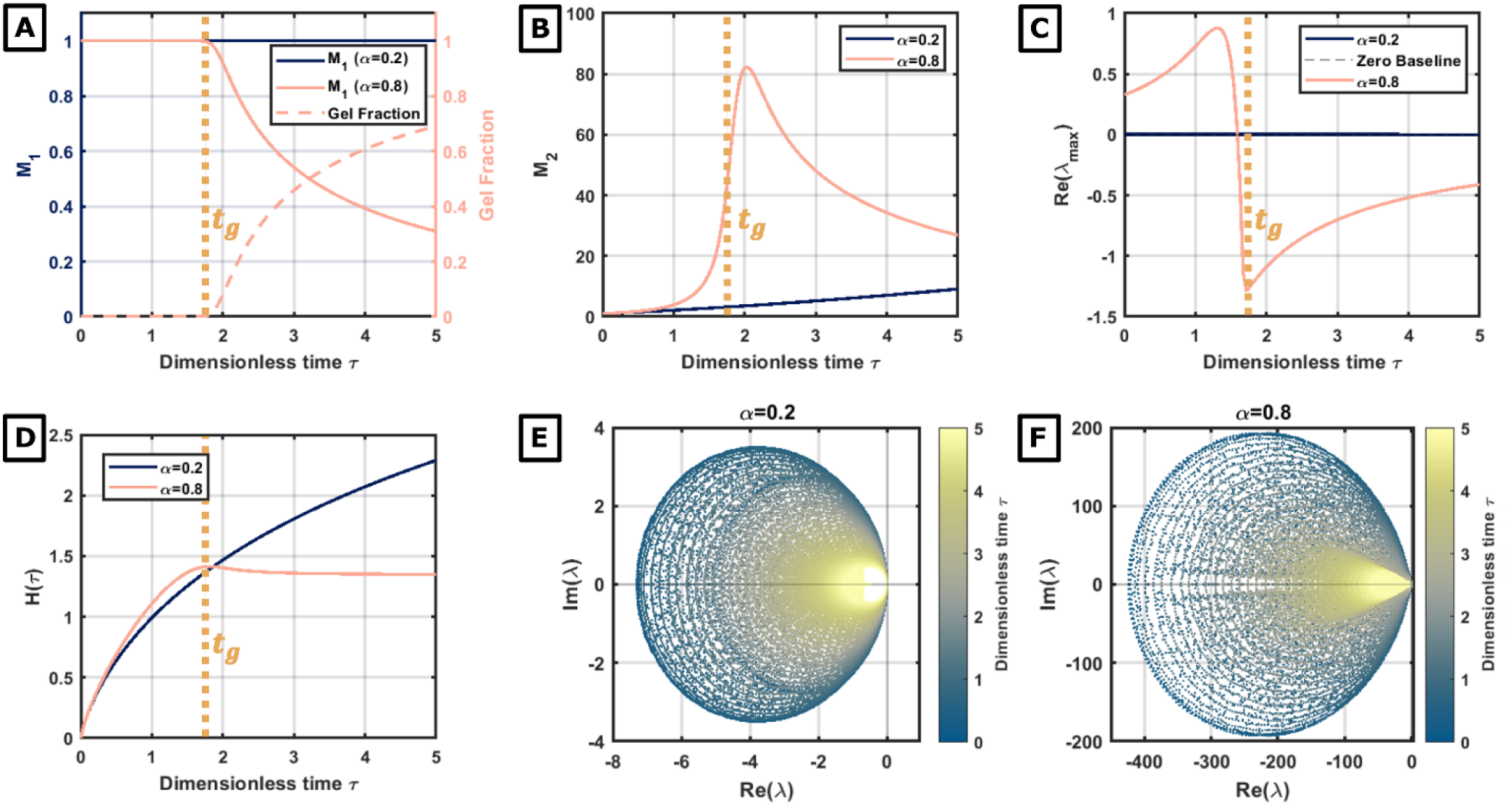
Dynamics of the nongelling and gelling regimes. (A) Evolution of the first moment (*M*_1_) for α = 0.2 and α = 0.8, and the gel fraction at α = 0.8. (B) Second moment (*M*_2_) evolution, indicating polydispersity. (C) Real part of the maximum eigenvalue (Re­(λmax)). The transition from positive to negative values indicates a shift in the dynamic stability of the population during the phase transition. (D) Evolution of structural parameter *H*(τ). Eigenvalue maps in the complex plane (Im­(λ) vs Re­(λ)) over time (color coded) for α = 0.2 (E) and α = 0.8 (F).

The dynamics change drastically in the gelling regime (α = 0.8). As the system approaches the critical gelation time *t*_g_, the second moment *M*_2_ diverges, and the first moment *M*_1_ subsequently exhibits mass loss (Figure 2A–B). Coinciding with this singularity (Figure 2C, pink line), Re­(λ_max_), which is already positive, undergoes a macroscopic excursion to a maximum peak, signaling the height of dynamic instability. Following the formation of the gel, Re­(λ_max_) drops sharply into negative values. This identifies the gel phase as a dynamically distinct state where the remaining sol population returns to a locally stable regime.

The structural complexity is tracked via Shannon entropy *H*(τ) (Figure 2D). In subcritical conditions (α = 0.2), blue, *H*(τ) grows monotonically as the distribution broadens indefinitely toward a polydisperse sol. In supercritical condition (α = 0.8), on the other hand, *H*(τ) saturates and decreases postgelation as mass “orders” into a macroscopic cluster, limiting the informational disorder of the remaining.

The structural difference between the two regimes is further highlighted by the cluster size distributions *c_p_* (*t*) (Figure 3). For α = 0.2, the distribution broadens over time but retains an exponential cutoff, characteristic of nongelling systems. In contrast, for α = 0.8, the distribution develops a power-law tail that extends to the cutoff scale *p* as *t* → *t_g_*, facilitating the formation of the macroscopic cluster. The observations in Figures 2 and 3 are not an artifact of initial particle monodispersity: polydisperse initial particle size distributions yield qualitatively the same observations (Figure S3).

**Figure 3:**
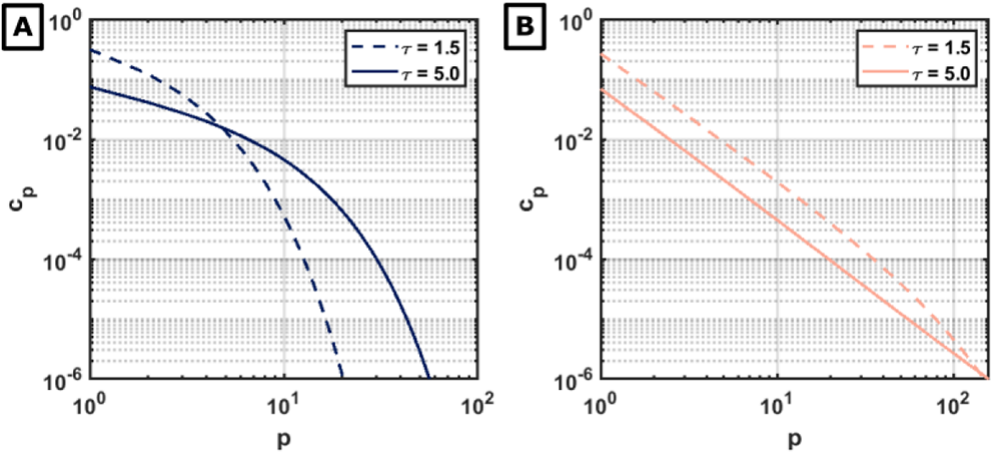
Cluster size distributions *c*_*p*_ at intermediate (τ = 1.5) and late (τ = 5.0) times, comparing the nongelling (α = 0.2) (A) and gelling (α = 0.8) regimes (B).

To ensure that the observed spectral instability is a specific signature of gelation and not a numerical artifact of growing cluster sizes, we analyze the physically motivated Smoluchowski diffusion-limited kernel (*D_f_* = 1.8). This kernel describes realistic aggregation in the continuum limit and is rigorously known to be mass-conserving (ω = 0).

The results, summarized in Figure 4, provide an important negative control. Despite the significant evolution of the system—evidenced by the growth of polydispersity *M*_2_ and Shannon entropy (*H*)—the spectral stability is perfectly preserved. The Jacobian spectrum remains strictly confined to the left half-plane (Figure 4D), and Re­(λ_max_(*t*)) remains vanishingly small (≈ 0) for all times (Figure 4C). Unlike the gelling case, there is no “leakage” of eigenvalues into the unstable right half-plane.

**Figure 4:**
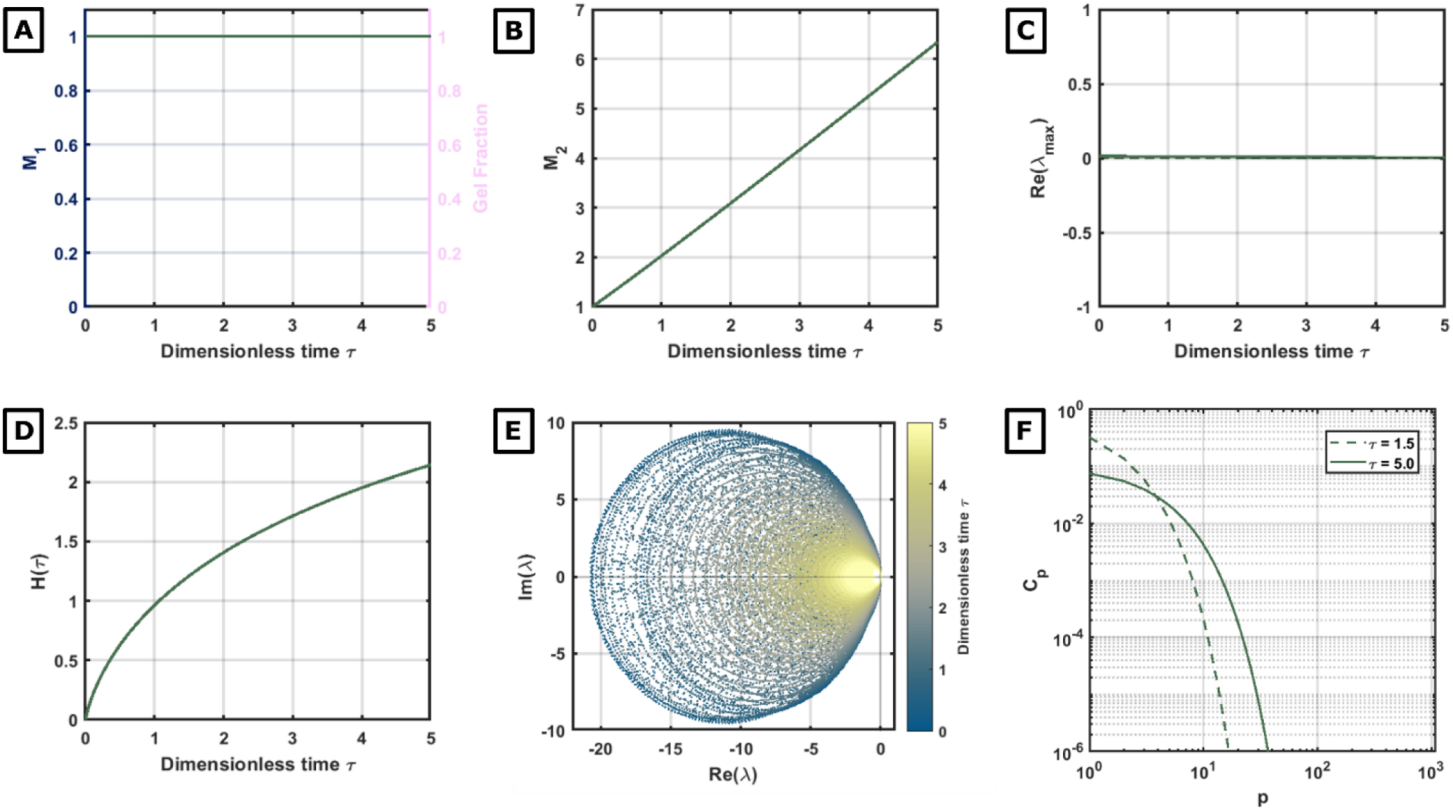
Dynamics of the nongelling Smoluchowski diffusion-limited kernel (***D* *_f_*** = 1.8). (A) Evolution of the first moment *M*_1_ (blue), which remains strictly conserved, and the gel fraction (orange), which stays at zero. (B) The second moment *M*_2_ grows monotonically but remains finite, contrasting with the divergence observed in gelling kernels. (C) The real part of the maximum eigenvalue, Re­(λ_max_), remains vanishingly small (≈0), indicating the persistence of dynamical stability. (D) Evolution of the Shannon entropy *H*(τ). (E) The full instantaneous spectrum of the Jacobian in the complex plane; all eigenvalues are confined to the stable left half-plane (Re­(λ) ≤ 0). (F) Snapshots of the cluster size distribution *c_p_* for intermediate (τ = 1.5) and late (τ = 5.0) times.

This confirms that our spectral framework is robust: it correctly detects instability in gelling kernels while avoiding false positives in physically growing but nongelling systems. The positive eigenvalues observed for α > 1/2 are therefore not trivial consequences of cluster growth, but markers of the catastrophic breakdown of the mean-field description associated with gelation.

## Conclusions

In summary, we demonstrate that kinetic gelation corresponds to a loss of local dynamical stability of the Smoluchowski flow, rather than solely to a moment-based singularity. This finding bridges the historical divide between the thermodynamics of nucleation (governed by the reversible Becker–Döring equations)^21^ and the kinetics of polymerization (governed by the irreversible Smoluchowski coagulation equation).^22^

In the nucleation limit, a positive Jacobian eigenvalue is the rigorous mathematical signature of a system crossing a free-energy barrier. Here, we show that the runaway growth of multiplicative kernels exhibits the same spectral topology. This commonality unifies these distinct physical processes—thermodynamic barrier crossing (which in colloidal systems is modulated by the interaction strength)^5,6^ and kinetic runaway—under a single dynamical principle: the onset of a cluster phase transition is signaled by a “dynamic divergence” of the stability spectrum.

Beyond aggregation, this trajectory-based framework generalizes to various evolving chemical systems. By focusing on the local response to perturbations rather than difficult–to–measure macroscopic signals, it identifies phase transitions through a clear spectral signature: the emergence of positive eigenvalues in the Jacobian. This redefines such transitions as a fundamental loss of local stability rather than a late-stage change, offering a robust, predictive tool for diagnosing kinetic runaway in transient, far-from-equilibrium processes.

## Supplementary Material



## Notes

Convergence analysis and finite-scale effects; evaluation of initial polydispersity on gelling regimes; cumulative Lyapunov growth profiles; mathematical derivations for time-dependent linearization and stability analysis (PDF)

## References

[1] Smoluchowski M. V. (1916). Drei Vortrage
uber Diffusion, Brownsche Bewegung und Koagulation von Kolloidteilchen. Phys. Z.

[2] Friedlander, S. K. Smoke, Dust, and Haze: Fundamentals of Aerosol Dynamics; Oxford university press: New York, 2000.

[3] Aldous D. J. (1999). Deterministic
and stochastic models for coalescence (aggregation and coagulation):
A review of the mean-field theory for probabilists. Bernoulli.

[4] Zaccone A., Wu H., Del Gado E. (2009). Elasticity of arrested short-ranged attractive colloids:
Homogeneous and heterogeneous glasses. Phys.
Rev. Lett..

[5] Zaccone A., Crassous J. J., Ballauff M. (2013). Colloidal gelation with variable
attraction energy. J. Chem. Phys..

[6] Zaccone A., Winter H. H., Siebenbürger M., Ballauff M. (2014). Linking self-assembly,
rheology, and gel transition in attractive colloids. J. Rheol..

[7] Leyvraz F. (2003). Scaling theory
and exactly solved models in the kinetics of irreversible aggregation. Phys. Rep..

[8] Lushnikov A. A. (1978). Coagulation
in finite systems. J. Colloid Interface Sci..

[9] Ernst M. H., Ziff R. M., Hendriks E. (1984). Coagulation
processes with a phase
transition. J. Colloid Interface Sci..

[10] Hendriks E. M., Ernst M. H., Ziff R. M. (1983). Coagulation
equations with gelation. J. Stat. Phys..

[11] Van
Dongen P., Ernst M. (1986). On the occurrence of a gelation transition
in Smoluchowski’s coagulation equation. J. Stat. Phys..

[12] Van
Dongen P., Ernst M. (1988). Scaling solutions of Smoluchowski’s
coagulation equation. J. Stat. Phys..

[13] Rouwhorst J., Ness C., Stoyanov S., Zaccone A., Schall P. (2020). Nonequilibrium
continuous phase transition in colloidal gelation with short-range
attraction. Nat. Commun..

[14] Rouwhorst J., Schall P., Ness C., Blijdenstein T. B. A., Zaccone A. (2020). Nonequilibrium master kinetic equation modeling of
colloidal gelation. Phys. Rev. E.

[15] Filbet F., Laurençot P. (2004). Numerical simulation of the Smoluchowski
coagulation
equation. SIAM J. Sci. Comput..

[16] Drake, R. L. A general mathematical survey of the coagulation equation; Pergamon Press, 1972.

[17] Sandkühler P., Lattuada M., Wu H., Sefcik J., Morbidelli M. (2005). Further insights
into the universality of colloidal aggregation. Adv. Colloid Interface Sci..

[18] Lazzari S., Nicoud L., Jaquet B., Lattuada M., Morbidelli M. (2016). Fractal-like
structures in colloid science. Adv. Colloid
Interface Sci..

[19] Goldhirsch I., Sulem P.-L., Orszag S. A. (1987). Stability and Lyapunov
stability
of dynamical systems: A differential approach and a numerical method. Phys. D.

[20] Coddington, E. A. ; Levinson, N. Theory of Ordinary Differential Equations; McGraw–Hill: New York, 1955.

[21] Duncan D. B., Dunwell R. M. (2002). Metastability in the classical, truncated
Becker–Döring
equations. Proc. Edinb. Math. Soc..

[22] Shaw S., Cademartiri L. (2013). Nanowires
and Nanostructures that Grow like Polymer
Molecules. Adv. Mater..

