# Supplementary material for: Gelation as a Dynamical Instability of the Smoluchowski Flow
Source: J Phys Chem B. 2026 Apr 14;130(16):4467–72. doi: 10.1021/acs.jpcb.6c01583 (PMC13112337; doi:10.1021/acs.jpcb.6c01583)
Supplement: Supplementary file 1 [file jp6c01583_si_001.pdf]

GELATION AS A DYNAMICAL INSTABILITY OF THE SMOLUCHOWSKI FLOW

*MANUEL DEDOLA†, LUDOVICO CADEMARTIRI†**

† Department of Chemistry, Life Sciences and Environmental Sustainability, University of Parma, Parco Area delle Scienze 17 A, Parma, Italy

** Author to whom correspondence should be addressed: ludovico.cademartiri@unipr.it*

SUPPORTING INFORMATION

CONVERGENCE ANALYSIS

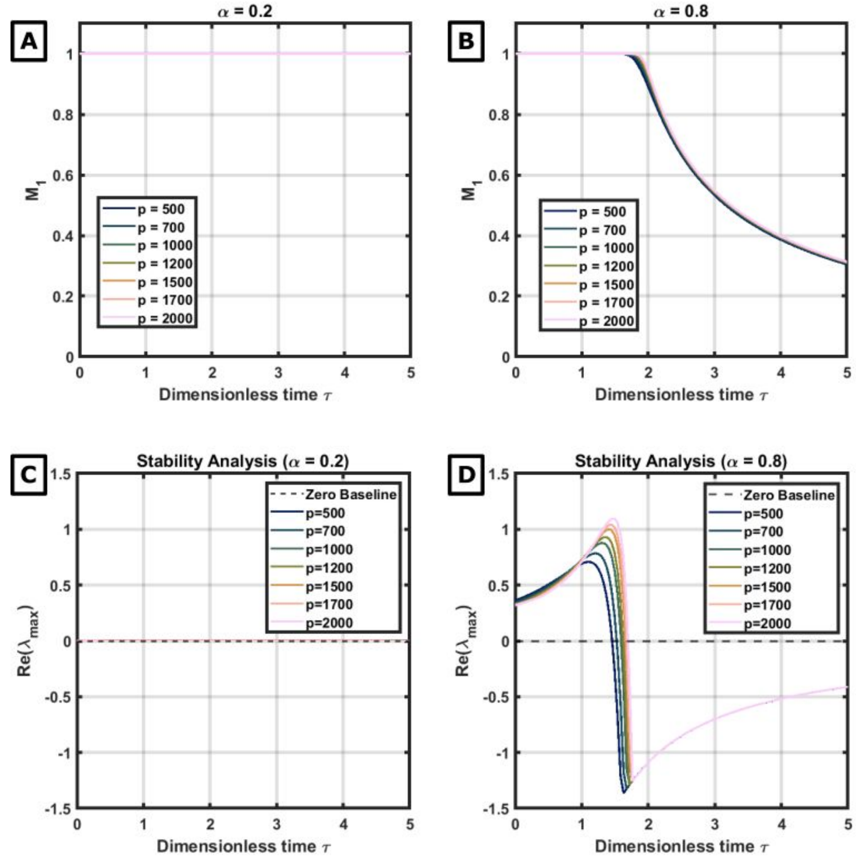

Figure S1. Finite-scale convergence. (A) First momentum $M_1(t)$ distribution for multiplicative kernel $K = (ij)^\alpha$ and $\alpha = 0.2$. **(B)** First momentum $M_1(t)$ distribution for multiplicative kernel $K = (ij)^\alpha$ and

$\alpha = 0.8$. **(C)** Stability analysis for multiplicative kernel $K = (ij)^{\alpha}$ and $\alpha = 0.2$. **(D)** Stability analysis for multiplicative kernel $K = (ij)^{\alpha}$ and $\alpha = 0.8$.

EFFECT OF POLYDISPERSITY

The polydisperse start uses an exponential distribution of particles ($c_p = e^{\lambda p}, \lambda = 0.5$). It is divided by its own mass, this ensures that the starting concentration is exactly 1.0 units of mass in both cases.

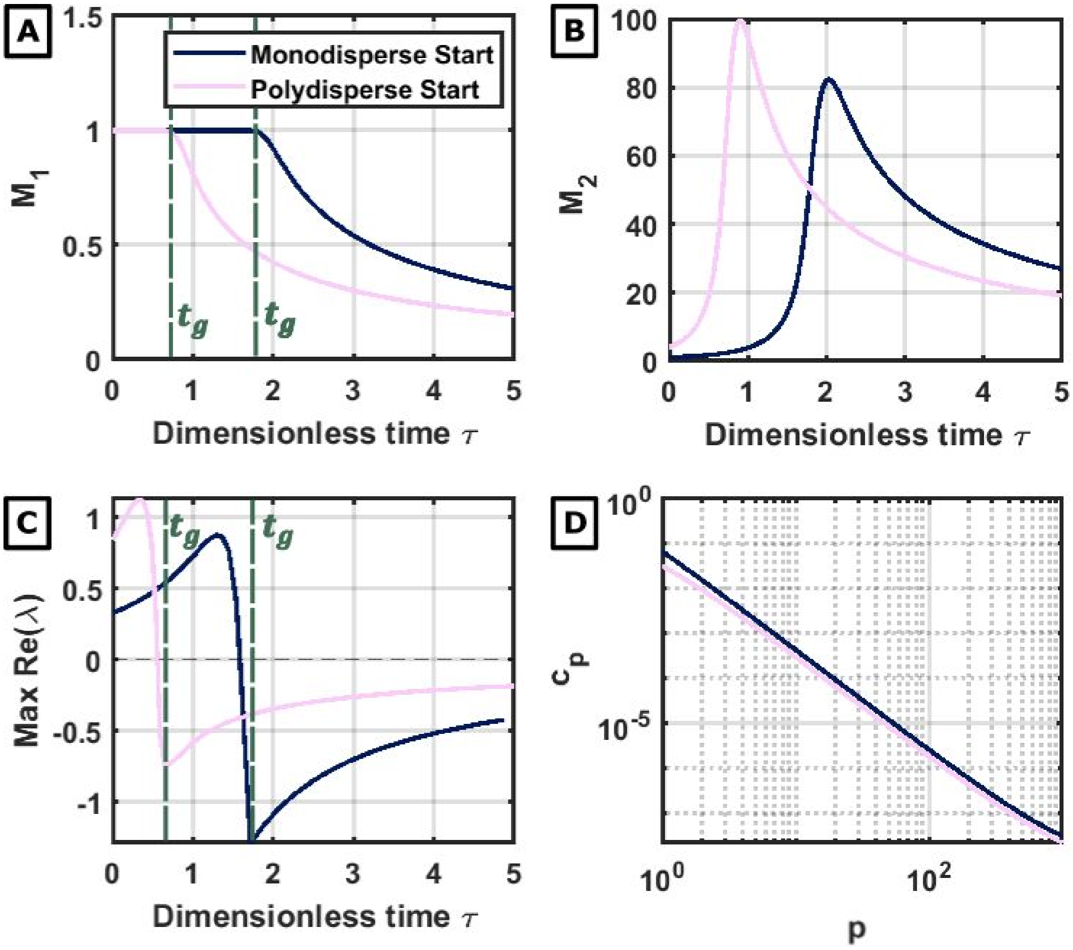

Figure S3. Dynamics of the gelling regimes $K = (ij)^{\alpha}$ with $\alpha = 0.8$ using monodisperse and polydisperse starting points. **(A)** Evolution of the first moment (M_1) **(B)** Second moment (M_2). **(C)** Real part of the maximum eigenvalue ($Re(\lambda_{max})$). The transition from positive to negative values indicates a shift in the dynamic stability of the population during the phase transition. (**D**) Cluster size distributions c_p at $\tau = 5.0$.

CUMULATIVE LYAPUNOV GROWTH

To quantify the total expansion of the flow, we define the Cumulative Lyapunov Growth **(CLG):**

$$\Lambda(\tau) = \int_0^{\tau} Re\big(\lambda_{max}(t')\big)dt'$$

which serves as a robust diagnostic for the onset of the cluster phase transition.

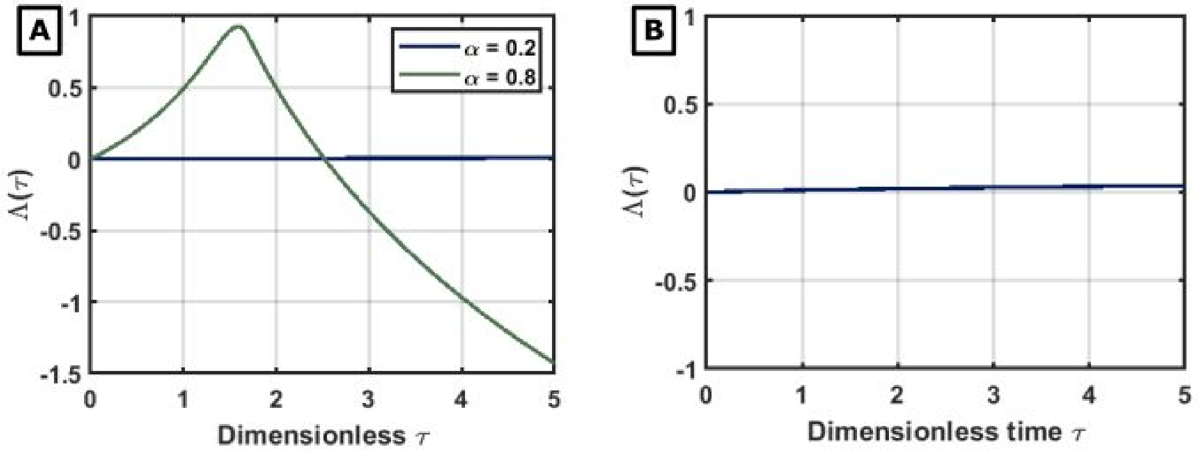

Figure S4 Cumulative Lyapunov Growth $\boldsymbol{\Lambda(\tau)}$. (A) Multiplicative kernel $K = (ij)^{\alpha}$ with $\alpha = 0.2$ and $\alpha = 0.8$. **(B)** Smoluchowski's kernel with $D_f = 1.8$.

LINEARIZATION AND STABILITY ANALYSIS

We investigate the local stability of the system by analyzing the evolution of infinitesimal perturbations $\delta\boldsymbol{c}(\boldsymbol{t})$ which physically represent spontaneous thermodynamic fluctuations or finite-size deviations from the mean-field limit.

Let $\boldsymbol{c}(\boldsymbol{t})$ denote the state vector of the system, governed by the nonlinear differential equation $\dot{\boldsymbol{c}} = \boldsymbol{F}(\boldsymbol{t})$.

We introduce a small perturbation $\delta\boldsymbol{c}(\boldsymbol{t})$ such that the perturbed state is given $\boldsymbol{c}(\boldsymbol{t}) + \delta\boldsymbol{c}(\boldsymbol{t})$. Substituting this expression into the governing equation and performing a Taylor series expansion truncated at the first order, we obtain:

$$\frac{d}{dt}(\boldsymbol{c} + \delta\boldsymbol{c}) \approx \boldsymbol{F}(\boldsymbol{c}) + \boldsymbol{J}(\boldsymbol{c})\delta\boldsymbol{c} \tag{S1}$$

By subtracting the equation for the unperturbed flow, equation $\dot{\boldsymbol{c}} = \boldsymbol{F}(\boldsymbol{t})$, from Eq. S1, we isolate the linear evolution equation for the perturbation:

$$\delta\dot{\boldsymbol{c}} = \boldsymbol{J}(\boldsymbol{c})\delta\boldsymbol{c} \tag{S2}$$

Here, $\boldsymbol{J}(t)$ represents the $p \times p$ Jacobian matrix evaluated along the trajectory. Its elements, $J_{ij}(t)$, describe the sensitivity of the rate of change of the i-th component with respect to the j-th variable:

$$J_{ij} = \frac{\partial \dot{c}_i}{\partial c_j} \tag{S3}$$

Since the Jacobian $\boldsymbol{J}(t)$ is time-dependent, the stability analysis requires a local approach. We adopt the *frozen-time analysis* framework, which assesses the instantaneous tendency of perturbations to grow or decay. At any fixed instant t, we treat the coefficients of the linearized system as momentarily constant (quasistationary approximation). Under this assumption, the general solution of the evolution of the perturbation $\delta\boldsymbol{c}(\boldsymbol{t})$ is a linear combination of all instantaneous eigenmodes:

$$\delta\boldsymbol{c}(\tau) = \sum_{i=1}^{p} a_i \, e^{\lambda_i \tau} \, \boldsymbol{v_i} \tag{S4}$$

where τ represents a local time scale relative to the frozen instant t. Substituting this ansatz into the frozen-coefficient version of Eq. S2 leads to the instantaneous eigenvalue problem:

$$\boldsymbol{J}(\boldsymbol{t})\boldsymbol{v} = \lambda_i \boldsymbol{v} \tag{S5}$$

Here, λ_i represents the set of instantaneous eigenvalues and $\boldsymbol{v}$ the corresponding eigenvectors. Since the general solution is a superposition of these modes, the long-term behavior of the perturbation is dominated by the eigenvalue with the largest real part. Consequently, if $Re(\lambda_{max}(t)) > 0$, the flow is locally expansive, providing a robust diagnostic for the onset of dynamical instability (gelation).
